# Uncovering the Value of a Historical Paper-Based Collaborative Artifact: The Nursing Unit's Kardex System

**DOI:** 10.3389/fdgth.2020.00012

**Published:** 2020-08-07

**Authors:** Austin F. Mount-Campbell, Kevin D. Evans, David D. Woods, Esther Chipps, Susan D. Moffatt-Bruce, Kashvi Patel, Emily S. Patterson

**Affiliations:** ^1^Value Institute, Christiana Care Health System, Newark, DE, United States; ^2^School of Health and Rehabilitation Sciences, College of Medicine, The Ohio State University, Columbus, OH, United States; ^3^Department of Integrated Systems Engineering, The Ohio State University, Columbus, OH, United States; ^4^College of Nursing, The Ohio State University, Columbus, OH, United States; ^5^Wexner Ohio State University Medical Center, Columbus, OH, United States; ^6^Department of Surgery, College of Medicine, The Ohio State University, Columbus, OH, United States; ^7^Royal College of Physicians and Surgeons of Canada, Ottawa, ON, Canada; ^8^College of Business, The Ohio State University, Columbus, OH, United States

**Keywords:** decision making, Health information-technology (HIT), computer-supported collaborations, qualitative methods, system design

## Abstract

We identify useful functions and usability characteristics of a historical cognitive artifact used by nurses working in a hospital unit, the Kardex. By identifying aspects of a widely used artifact, we uncover opportunities to improve the usefulness of current systems for hospital nurses. We conducted semi-structured interviews with registered nurses about their prior experience with the Kardex. Questions included what elements of the Kardex are missing from their current electronic support. Memos were generated iteratively from interview transcript data and grouped into themes. Eighteen nurses from multiple clinical areas participated and had a median of 25–29 years of nursing experience. The themes were: (1) a status at a glance summary for each patient, (2) a prospective memory aid, (3) efficiency and ease of use, (4) updating information required to maintain value, (5) activity management, (6) verbal handover during shift-to-shift report, (7) narrative charting and personalized care, and (8) non-clinical care communication. Implications for digital support are to provide immediate, portable access to a standardized patient summary, support for nurses to manage their planned activities during a series of shifts, provide unstructured text fields for narrative charting, and to support adding informal notes for personalized care.

## Introduction

With digital health information technology, we can design innovative features by analyzing the functions of historical paper-based artifacts and “workarounds” (1) to the intended use of digital technology. In this paper, we analyze the functionality of a historical paper-based artifact used on many nursing units in hospitals in the United States before the introduction of the electronic health record, the Kardex. Nurses often report sadness due to the loss of the Kardex. The defining elements of the Kardex include that it was a formally sanctioned paper-based information system for nurses working in a hospital unit to support situation awareness and activity planning during a patient's stay on the unit. Nurses used the Kardex in many hospitals, typically formatted as one piece of card stock per patient containing structured summaries, handwritten in pencil. Multiple nurses updated the summaries across the course of stay. This “nursing Kardex” is distinct from a “medication Kardex” used collaboratively with pharmacists to track pharmacy-labeled pharmaceutical medications ordered for each patient on the unit (2). Our analysis is based on semi-structured interviews with a convenience sample of registered nurses with substantial historical experience using the Kardex in hospital settings to provide bedside nursing care.

In many settings, paper forms [e.g., patient admission forms in hospitals, (3)], paper handouts [e.g., financial advisors meeting with clients, (4)], printed documents [e.g., academic supervisors reviewing papers with their students, (5)], and handwritten notes on paper [e.g., taken by policemen, (6)] have persisted in the presence of digitization and technological innovation. In some cases, the use of paper persists despite explicit organizational incentives or sanctions intended to discourage use [e.g., paper charts used by physicians in outpatient care, (7); handwritten patient summaries used by nurses in hospitals, (8, 9)].

In the United States, there has recently been extensive digitization of the information infrastructure in hospital settings. As part of this transformation, nurses are arguably the most adaptive and resilient care providers; they are also one of the primary users of paper-based cognitive artifacts (10). Despite the digitization of healthcare, nurses are using extensive handwritten notes, which nurses refer to as “brains” or “report sheets.” These handwritten notes on structured sheets organize information scattered throughout electronic resources; nurses use these cognitive artifacts to support care planning, clinical immediacy, and handover reports (8). While the benefits of the “brains” artifact have been well-documented (9), many are interested in replacing them with electronic systems.

Understanding the benefits and limitations of the Kardex, a previously widely used cognitive artifact, has implications for the modification of current hospital environments. As one option, the Kardex could be reintroduced, at least in part, to replace lost functionality. More appealing to many would be to incorporate the lost functionality into the digital infrastructure through integration into the electronic health record; this approach would likely better protect patient privacy and storage security for information with sensitive patient data. Finally, the use of “brains” by nurses could be formally encouraged and standardized across a unit to replace lost collaborative functionality. Beyond functionality, administrators and information technology may benefit from understanding what aspects of a formally designed and sanctioned system (as opposed to paper-based artifacts whose use is actively discouraged) inspired such unprompted and widespread loyalty years to decades after removal.

Healthcare, similar to many other domains, has been undergoing widespread digitization of the underlying infrastructure for care provision. In 2006, fewer than 10 percent of the hospitals in the United States used electronic health records (EHRs). Following the executive order for a 10-year development of EHRs and the Health Information Technology for Economic and Clinical Health Act of 2009, this increased to 93–99% of all US hospitals by 2017 (11).

Following the implementation of the electronic health record, paper-based artifacts have persisted; cognitive artifacts are essential to nursing handovers, especially those on paper (12). These artifacts provide a narrative format that is centrally important in quickly, effectively, and empathetically, as well as communicating patient stories in a format that is easy to remember (13). Also, the schema for experts makes it easier to work with recognizable archetype narratives with which they have more experience (14). McLane et al. (15) provided a detailed description of the functional usage and value of paper-based records for nurses in the post-EHR implementation environment. Important elements include providing a quick reference for handwritten information in an organized structure, a patient's status at a glance' overview, care planning, and clinical immediacy (15).

The Kardex was a formal “blunt-end” artifact that provided patient information to nurses and assistants on a nursing unit. Although not part of the formal medical record, it was utilized exclusively by nurses and related staff at most hospitals in the United States in prior years and has continued to be used in a small number of hospitals since the widespread adoption of EHRs. Although a formal system, the Kardex was designed for the “sharp-end” (16) in that nurses providing bedside care managed the system, and the Kardex was typically discarded or placed in the patient paper chart upon patient discharge from the unit. With the EHR implementation being widespread, the Kardex was phased out by administrators to avoid redundant systems and also to achieve the goal of having a paperless hospital. Removing the formally sanctioned Kardex has contributed to the expansion of the reliance on an informal, unsanctioned workaround artifact (the “brains”) (8, 9). Staggers et al. (12) characterized the widespread use of the “brain” as a workaround artifact by nurses as a central aspect of “hidden lives” (12).

The traditional Kardex contained information with printed labels in an organized layout with handwritten notes. Much of the information was available in the patient chart, including patient identifiers, laboratory results, orders, diet, areas of concern, diagnosis, and narratives about patient care before and during the hospital stay. Information uniquely captured in the Kardex, but not elsewhere, included handwritten notes taken during and to support shift change report. These notes included sensitive comments about the patient, such as about potential substance abuse and about family members such as the possibility of domestic violence.

We report the findings of a semi-structured interview study designed to answer this research question: *What useful functionality, and in particular collaborative affordances, was provided by the Kardex for registered nurses in hospitals?* We follow the definition of a collaborative affordance as “properties of physical and digital artifacts that afford collaborative activity in a specific context” [(17), p. 2]. To address this question, we asked questions contrasting the functionality, benefits, and challenges of the Kardex with both electronic health records and paper-based handwritten notes (a.k.a., “brains”). We included a question asking participants to sketch the layout of the Kardex and what information was included in which section. We summarize and discuss our insights regarding the affordances of the Kardex as well as the insights regarding conducting a historical study of a paper-based artifact to spur innovation with digital technology.

## Materials and Methods

This research complied with the American Psychological Association Code of Ethics and was approved by the Institutional Review Board at Ohio State University. Informed consent was obtained from each participant.

The inclusion criteria were being licensed as a registered nurse, having experience working with a nursing Kardex in an adult or inpatient pediatric care unit, and a willingness to participate. There were no exclusion criteria.

Semi-structured interviews were conducted face-to-face or over the phone by a single investigator, with the questions detailed in Table 1. A moderator's guide supported real-time note-taking, and interviews were digitally audio-taped and transcribed. When the participants sketched the Kardex layout, a picture was taken of the handwritten sketch and shared digitally. Interviews ranged from 18 min and 28 s to 59 min and 4 s, with an average of 39 min and 12 s and a standard deviation of 9 min and 59 s.

**Table 1:** Semi-structured interview topics and questions.

**Topic**	**Questions**
Kardex	During the era of the Kardex, when did you use the Kardex system during your shift? What information were you accessing when using the Kardex? How was the Kardex organized? What information was on the Kardex? Missing from the Kardex? What was your favorite aspect of the Kardex? Least favorite? Did you use other paper forms in conjunction with the Kardex? Explain. Did you use a pen or pencil to write information on the Kardex? Was your task-list on the Kardex, “brain,” or neither? What happened to the Kardex after the patient was discharged? Do you have anything else to share about the Kardex?
Transition to EHRs	What do you think was lost when transferring from paper-based records to electronic health records? What new functionality was provided that was not available with the paper-based system for documentation? Comparing the Kardex to the EHR, what would you change? What would you bring back from the Kardex? How has the EHR to Kardex transition affected your job process, workflow, or job description?
Handwritten personal notes	Did your paper notes or “brains” (or report sheet) change when the Kardex was gone? Were they more or less important? How did the Kardex layout differ from the layout of your notes? What information did you handwrite that was not in the EHR? Or was not conveniently located in the EHR? How did the structure and order of your personal handwritten notes differ from the EHR? How did you organize your handwritten artifact and why? Please sketch how it was organized? Did you ever share your handwritten notes with other nurses?
EHR experience	What is the best part of the EHR? The worst? What would you change about the EHR if you could? Do you have anything else you would like to tell me about the EHR and Kardex systems?
Handover	What tools do/did you use to aid your handoff? Are they integrated into the EHR?
Design ideas	Do you have any design ideas for a system that would aid you in your daily job tasks?
Anything else?	Is there anything else you want to tell me about information or data management in your work as a nurse?

The participants were a convenience sample based upon recruitment by two nursing managers from two hospitals in the Midwest and Southwest and personal networks of the research investigators. We provided no financial compensation for participation. We conducted interviews until theoretical saturation was achieved, as defined by no new insights or emerging themes in the last two or more participant interview sessions (18).

We used a bottom-up qualitative approach in the analysis, which was also informed by concepts from the computer supported cooperative work literature, human factors literature, and health informatics literature. During the first iteration of analysis, the transcripts were grouped roughly into three sections, which reflected the ordering of the questions: (1) while using the Kardex, (2) when transitioning from the Kardex to the EHR and personal handwritten notes (“brains”), and (3) after the Kardex was removed. Grounded theory was used to let the data drive the emergent themes (19). Throughout the coding process, a constant comparison method was used in generating the themes (20). Memos (21) were handwritten in the margins; in this case, memos were textual mini-summaries of major findings that were later grouped to make an initial list of themes. The exact memos written on the margins are included in the results for each final theme, with similar memos grouped together in a theme. Key text from the verbatim transcript generated by a professional transcription service was highlighted with a color associated with the memo on the printed transcript. All of the memos were generated by a single investigator, a PhD student with prior experience conducting qualitative analysis and who had taken coursework in qualitative methods and ethnographic methods. This investigator generated the initial themes, which were vetted and modified by the entire investigative team, which included clinical experts. A second investigator reviewed a random sample of 10% of the parsed transcripts and applied codes from a codebook containing descriptions and examples for the initial five themes. An inter-rater reliability Kappa score was calculated to compare the codes from the two raters. A third investigator re-analyzed all of the transcripts in order to identify potential additional themes and decompose themes with more than one concept into additional themes. Several themes were renamed to be more specific and additional themes added to the codebook. All themes were discussed by all investigators until consensus was achieved and the resulting eight themes were reported, along with the original memos and representative quotes from the transcripts. When available, the specific findings for each theme were compared with published results from direct observational studies of nursing personnel using either the paper-based Kardex or an electronic health record.

After we identified the eight themes, validation interviews were conducted with three additional nurses. These nurses were doctorally trained nursing colleagues with prior experience using the Kardex, one of whom served as a pilot participant in the original interviews. During this validation interview, the eight themes were described. All of the participants were first asked to provide alternative names for the themes, emphasizing that descriptions that would resonate with nursing personnel were particularly important to capture. These alternatives were included in modified descriptions of the reported themes. Next, the participants were asked to rank the themes from one to eight in relation to “how the Kardex used to be used,” with one being the most relevant and eight being the least relevant. In the event that the participant did not feel that any of the themes were relevant, they were asked not to provide any rank for those. Third, a series of semi-structured interview questions were asked:

1. Are there any themes you feel are missing regarding the Kardex?
2. What aids do you currently use to record/remember information that is not documented in the electronic health record?
3. Is there anything about the electronic health record that works better than the Kardex did?
4. Is there anything else you would like to share on this topic?

## Results

### Demographics of Registered Nurse Interview Participants

As shown in Table 2, 18 experienced registered nurses participated in the interviews. The range of prior experience with the Kardex was 1–4 to 25–29 years, with a median value of 10–14 years.

**Table 2:** Demographics of interviewed participants.

**No**.	**Clinical unit experience**	**Nursing experience (yr)**	**Kardex experience (yr)**	**EHR experience (yr)**
1	Surgery	30–34	15–19	30–34
2	Critical care; Med-surg	30–34	15–19	1–4
3	Critical care; Med-surg	40–44	20–24	5–9
4	Med-surg	25–29	15–19	10–14
5	Mental health	30–34	25–29	25–29
6	Pediatric critical care; Post-partum care	35–39	10–14	5–9
7	Critical care	30–34	25–29	5–9
8	Pediatric critical care	15–19	5–9	15–19
9	Progressive care	45–49	10–14	1–4
10	Surgery	30–34	20–24	1–4
11	Critical care	20–24	15–19	10–14
12	Critical care; Med-surg	25–29	5–9	15–19
13	Progressive care	20–24	1–4	10–14
14	Med-surg	15–19	5–9	15–19
15	Surgery	10–14	10–14	5–9
16	Med-surg	5–9	5–9	1–4
17	Pediatric surgery	15–19	10–14	5–9
18	Pediatric critical care	5–9	5–9	5–9

*EHR, Electronic Health Record; Med-surg, medical surgical*.

### Eight Emergent Themes

We identified eight emergent themes during the analysis. The last three themes were originally grouped within the first five themes.

#### Theme 1: ‘Status at a Glance’ Patient Overview

The first theme regarded having a snapshot overview (or “status at a glance”) in one place. Nine of the study participants (9/18; 50%) had a related memo. The memos were: (1) overview, (2) snapshot, (3) all in one place, and (4) dashboard. Seven participants (7/18; 39%) used the Kardex most often at the beginning of the shift when they were planning what to do during the shift before they had assessed any patient. Six participants (6/18; 33%) said that they accessed the Kardex often, checking in frequently throughout the shift for updates and refreshing their “overview” understanding of the patient. Representative quotes included, “The Kardex was elegant in terms of everything was centralized and that part I liked in terms of not missing critical activities,” “only had the information that you needed,” and “snapshot summary.”

Our findings are similar to a prior study of the information included in the Kardex based upon real-time observation of the system in use (22). In that study, the Kardex included for each patient the name, age, status regarding whether or not to resuscitate the patient (Do Not Resuscitate status), marital status, religious affiliation, allergies, medical diagnoses, emergency contact numbers, permitted activities, and functional limitations. Our findings differed from this prior study in that the study participants reported that the following information about orders, which they observed, was not always included: medications, treatments, diet, IV therapy, tests, procedures. In addition, our study participants did not report that consultations were included.

#### Theme 2: Prospective Memory Aid

We grouped four of the original memo groups because they dealt with supporting memory in regards to information and activities, including (1) notes, (2) scribble notes, (3) blank sheets, and (4) “brains.” All but one study participant (17/18; 94%) described how handwritten notes, “brains,” were used to support remembering information. Representative quotes included: “I don't trust my memory, I'm going to write it down on whatever I can write it down” and “A lot of what was on the Kardex used to put in this piece of paper that you carried around with you because you didn't carry the Kardex around and the paper-brains like helped you keep track of what you needed to do,” and “There's always going to be a need…to jot those things down.”

#### Theme 3: Efficiency and Ease of Use

The Kardex was viewed as simple, efficient, and easy to use, which related to memos titled: (1) simple and (2) efficient (and time-intensive, time wasted). All of the participants who were willing to sketch the layout (8/18; 44%) used a single box to depict a card for an individual patient. The sketches were helpful for study participants to describe the format in general, but not detailed enough to do an information content analysis by location. In some cases, the back side of the card was used for information, including medication information or notes. Some of the sketches described card stock where related tools, such as the medication Kardex for the patient, was on a ‘pullout card’ in a standardized way.

The majority of the participants (13/18; 72%) contrasted the Kardex positively in comparison to the transition to the EHR and removal of the Kardex. In other words, the Kardex was comparatively described compared to the EHR as more efficient, less challenging to use, or better-organized. Regarding information in the Kardex, nearly half of the study participants (8/18; 44%) felt that nothing was missing from the Kardex concerning relevant information. On the other hand, six (6/18; 33%) felt that medication information was missing from the Kardex. In comparison, with the EHR, five participants (5/18; 27%) felt that more time was needed to gather information and that this task of gathering information did not add value. Nine participants (9/18; 50%) identified that the EHR enabled access to more types of care providers than the Kardex, which required physically going to a particular room on the unit where patients were not in the room. A representative quote for this theme is: “The Kardex you knew had all the information that you needed to know about the patient in a pretty small format. And now, I think with these computer systems, they almost give you too much access. It's just so overwhelming. I guess, for me, it would be nice if each hospital could lay out their computer Kardex to look like their old Kardex.” With a more efficient system used by all nurses on the unit, more time is available for care activities at the bedside and less time to handwriting information on a personal report sheet.

#### Theme 4: Updating

Two of the original memos, both stated negatively, indicated that the Kardex was not always updated. Therefore, this theme covers these memos: (1) updating issues, and (2) not up to date. Eight of the study participants (8/18; 44%) reported that the Kardex was not always updated. A representative comment was: “It was only as good as it was updated.”

Although it was not explicitly stated what tended to be updated or when during the interviews, some of the answers included information about updates. Mostly, mentions of updating the Kardex were provided in the context of discussing the Kardex supporting the shift-to-shift report and while erasing and adding tasks planned to be done during the coming work shift. None of the study participants described going to the nursing conference room solely to update the Kardex information. Instead, they suggested that handwritten notes (“brains”) carried by nurses would be where “jots” to remember to update information later were documented.

The insight that the Kardex was not always updated, as well as the inference that the EHR might be easier to update than the paper-based system in one location away from patient rooms, has not been confirmed to our knowledge in the published literature. Regarding electronic health records, one element of information insufficiently maintained on the Kardex was identified in a recent study of care teams. In this study, care teams, including nurses, in community health centers in Oregon and Washington provided care using electronic health records, and the findings described 10 unmet information needs. Their first reported unmet information need was that information about social determinants of health was inconsistent and infrequently updated in the electronic health record (23).

#### Theme 5: Activity Management

In all periods, including when using the Kardex, when transitioning from paper-based records to electronic health records, and in the post-Kardex era, the theme of managing planned tasks during a shift with a memory aid persisted. The format for tracking planned activities was a task list or a to-do list. Therefore, this theme covers these memos: (1) To-do list, and (2) task list. Most study participants (10/18; 55%) described how to create lists, task lists, or care plan activities as handwritten notes either based upon information in the Kardex, shift report, or the EHR. Several of the participants emphasized the importance of having personal information, which was always immediately accessible without returning to the Kardex to remember to do a planned task. Similarly, participants made comments regarding how heavy and thick Kardex cards, in combination with handwritten information in pencil using an eraser, supporting planning, and managing tasks over time across multiple shifts. Representative quotes for this theme include: “I still have dreams about the brain, you know that piece of paper and nightmares about losing it and not knowing what I'm doing on my patient,” and “We would have all kinds of lists, but at the end, I would make my own separate to do, or I would even time it out, like here's what I have to do at these times, so it really just helps my day.”

#### Theme 6: Verbal Handover During Shift-to-Shift Report

An emerging theme was support for the verbal handover during the shift-to-shift report. Many of the study participants described the importance of accessing the Kardex around the time of the shift change, which is also confirmed by a prior study that observed shift change meetings always taking place in the nursing conference room where the Kardex was stored (22). Five of our participants (5/18; 28%) described using the Kardex most often during the shift change. Both the historical Kardex and the handwritten sheets (“brains”) supported remembering what to say during the verbal handover and writing down notes from the verbal conversation to support work during the upcoming shift. Representative quotes for this theme include: “Kardexes were often used to guide shift-to-shift report,” “We would update it [Kardex] as we were taking report,” and “If you were doing a transfer of a patient, you would pull the Kardex in addition to having their record in front of you so you would have some of the key aspects to tell – when they had their surgeries when they had various procedures done.” At least one study participant commented that the concept of a “handover” was not common at the time when the Kardex was used and that more typically the verbal interaction between a nurse taking over the care of the same set of patients as the nurse who was ending a shift was referred to as “giving report” or “giving patient report.”

#### Theme 7: Narrative Charting and Personalized Care

The Kardex was perceived as giving nurses more time for personalization and face-to-face patient care than the current electronic health record design. Half of the study participants (9/18; 50%) described a loss of support for narrative charting when the Kardex was removed. The loss of narrative charting was explicitly noted as lost in modern electronic health record support, with a representative quote being “There's really not a lot of narrative charting.” This loss of a narrative when charting was stated along with a perception that the computerized support was negatively interfering with the nurses' relationship with the patient and ability to provide personalized care. Representative quotes for care provided with the support of electronic health records after the Kardex was removed included: “I often hear nurses say providing care based on the computer as opposed to providing care for the patient,” and “I think it's more looking at things today not necessarily the patient, but a machine.”

#### Theme 8: Non-clinical Care Communication

Some of our study participants identified that the non-permanent nature of writing in pencil on a card that was discarded when a patient was discharged on the unit and was not included in the official medical record, afforded the opportunity to include non-clinical information about a patient. This information was helpful to navigating interactions with the patient and family members, and particularly with respect to sensitive topics such as substance abuse and domestic violence. In addition to providing higher quality care, some of this information was important for nurses to personally protect themselves from harm while working. In contrast to the non-permanent nature of the Kardex, with the EHR, by design, there are more users for the entered data than nurses on a unit providing direct bedside care in the next few shifts and days. Additional users include other healthcare providers, billing specialists, quality improvement personnel, and researchers analyzing patients in sets rather than as individuals. The electronic health record data is typically accessible during lawsuits and at the request of patients, whereas the Kardex had no permanent record or access by patients or lawyers. Eight of the study participants (8/18; 44%) reported that the Kardex was the primary way that they communicated with nurses taking care of the patient in the future. A representative comment was: “Or, it could be something about the patient, like, he spits on you or bites or something like that, that again you wouldn't necessarily want to put on a medical record but you certainly would want to share with your colleague in terms of being able to provide safe care.”

### Validation Interview Results

The original descriptions of the eight themes are displayed in the first column in Table 3. After the description and explanation for each individual theme, the three experts provided the alternative labels provided in Table 3. Blank entries indicate that the participant was unable to provide an alternative label or was satisfied with the current label.

**Table 3:** Alternative names for eight emergent themes.

	**Alternative theme name**		
**Original theme name**	**Participant 1**	**Participant 2**	**Participant 3**
“Status at a Glance” patient overview	Snapshot	Patient snapshot/overview	Patient at a glance
Prospective memory aid	What I would do today	Checklist	
Lightweight	Portable, pocket notes	The brain	Pocket, portable
Updating	Accuracy		Out of date
Managing activities in time	To do list	Schedule	Task list
Supporting a verbal handover		Preparing for report	
Narrative charting		Whole person care	Loss of nurse-patient connection
Sharing sensitive information in a nonpermanent manner	Need to know but not to write	Confidential nurse-to-nurse communication	

In Table 4, the results of the rankings of the themes from most to least relevant are provided. The second participant did not feel that two of the themes were relevant at all to her prior experience with a particular Kardex system. All of the participants ranked the theme of “‘Status at a Glance’ Patient Overview” as the most relevant, and all of the participants ranked the theme of “Managing Activities in Time” as either the second or third most relevant theme. All three of the participants ranked “Lightweight” as either not relevant at all, the least relevant, or the second least relevant. The ranking on “updating” of being the least relevant by one participant may have been due to viewing the Kardex as difficult to update, which confirms the theme and perceptions from the interviews. One participant ranked the ability to share sensitive information between nurses without having a permanent record as the second most relevant element. In contrast, other participants rated that aspect as one of the least relevant ones.

**Table 4:** Rankings of relevance of findings for Kardex.

**Theme**	**Participant 1**	**Participant 2**	**Participant 3**
“Status at a Glance” patient overview	1	1	1
Prospective memory aid	2	2	6
Lightweight	7	0	7
Updating	3	0	8
Managing activities in time	3	2	3
Supporting a verbal handover	5	4	4
Narrative charting	4	3	5
Sharing sensitive information in a nonpermanent manner	6	5	2

*1, most relevant; 8, least relevant; 0, not relevant to how the Kardex used to be used*.

In Table 5, the responses to the semi-structured interview questions are summarized. All three participants identified that our themes did not capture the interdisciplinary communication support provided by the Kardex. One noted that therapists would view their Kardex, one pointed out that the interdisciplinary team would view their Kardex, and one stated that social services viewed their Kardex, but physicians did not. The negative aspect of lack of updating of the Kardex leading to outdated or in accurate information was confirmed, as well as a perception by two that the EHR better supported updating information and accessing up-to-date information. One participant identified a new negative aspect of the Kardex, which is that the paper Kardex card could be misplaced, and that this does not happen with electronic health records.

**Table 5:** Validation interview summarized responses.

**Question**	**Participant 1**	**Participant 2**	**Participant 3**
Are there any themes you feel are missing regarding the Kardex?	Flexibility	Shared by others such as therapists	None
What aids do you currently use to record/remember information that is not documented in the EHR?	None	Paper brains	None
Is there anything about the EHR that works better than the Kardex did?	Accuracy/updating	Could misplace Kardex but cannot lose EHR	Updating, communicating with the interdisciplinary team
Is there anything else you would like to share on this topic?	Doctors did not look at Kardex—it was nurses' and social services vs. everyone is on the EHR	Nothing	Nothing

*EHR, Electronic Health Record*.

## Discussion

Eight themes depicting useful functions of the historical Kardex system were identified from the semi-structured interviews with 18 RNs from a variety of hospital care settings. The eight themes of the Kardex with positive and negative aspects are presented in Table 6. Particularly positive aspects of the Kardex were that it provided a status at a glance summary of a patient in a narrative charting format, which was shared by all of the nurses on unit. The Kardex supported managing activities across nurses as well as across shifts, as well as sharing sensitive information which might not need to be documented in a formal chart. Regarding negative aspects, the primary drawback to the Kardex was that it was not always reliably updated, particularly for the pharmaceutical medication information. Except for one Kardex used in a Neonatal Intensive Care Unit, the system was not physically available at the patient's bedside and therefore updating it required traveling away from the patient to the area where it was stored. If they conducted the shift report update in a different location than where the Kardex was stored, typically, the Kardex was taken away from the usual location for the duration of the handover. During this time, typically, other nurses or clerks would delay written updates until after the nurses had completed their verbal interaction.

**Table 6:** Summary of positive and negative aspects of the Kardex themes.

**Theme**	**Positive aspect**	**Negative aspect**
Status at a glance summary for each patient	Provided a patient summary that did not require logging in to access	The nurse had to physically go to a room where the Kardex was stored to access the summary
Prospective memory aid	There was detailed information in a succinct format about patients who were receiving bedside care during a work shift, including ‘jots’ and highlights of noteworthy information	Not able to electronically schedule a reminder to do something because it is on paper
Efficiency and ease of use	Well-organized layout which was not overwhelming to scan	The nurse could not usually carry the card around in a scrub pocket, so it was not immediately accessible
Updating information required to maintain value	Information was updated at the beginning of a shift and during shift-to-shift report	Information was infrequently updated at other times; Medication information was inconsistently included
Activity management	Nurses could manage activities for multiple nurses across all the shifts that the patient was on the unit; each subsequent nurse could erase tasks written in pencil with an eraser without ripping the paper and replace with new tasks	Erasing tasks required an eraser and took time to do; Erased activities were not available later
Verbal handover during shift-to-shift report	Supported report in the nurse conference room where the Kardex was permanently located, with both the Kardex and the paper chart open and guiding the update about what key aspects to highlight from the nurse ending his or her shift while the nurse starting his or her shift wrote notes directly onto the Kardex card based on the verbal update	In some units where the Kardex was not stored at the bedside and it was not allowed to be moved, they could not easily do a bedside report or had to coordinate reports with other nurses
Narrative charting and personalized care	Narrative charting was useful for nurse to nurse communication across work shifts to provide bedside care for the next hours to days and did not require a nurse to turn his or her back to the patient when communicating or reduce time spent at the bedside assessing and caring for a patient	No negative aspects reported
Non-clinical care communication	Sensitive information was included, such as when a patient might be illegally abusing substances, be at risk of harm at home from a spouse, or when a nurse might be at risk of harm from aggressive actions from a patient or family member.	No negative aspects reported

These insights have implications for designing innovative digital support for nurses in hospitals. For electronic health records, innovative features could be added without substantial modification. These features include: (1) providing immediate, portable access to a standardized patient summary without having to log in, such as by having barcoded badge identifiers or fingerprint-based identification on a tablet or smartphone device, (2) augmenting existing support for individual nurses managing the planning of an individual shift to multiple nurses across all the shifts that a patient is cared for on the unit, including being able to personally remove, modify or add activities for the current shift, (3) providing unstructured text fields for narrative charting that can efficiently modify narratives from the same patient in past shifts or other patients with similar characteristics, while also making clear what was copied from elsewhere and what was documented recently, and (4) expanding existing support for informal notes about a patient to allow personal notes only viewable by an individual or designated small number of nurses providing care to a patient.

A prior finding (24) was that the Kardex supported a workaround process to allow nurses to administer medications to patients based on a verbal order before official entry by the physician into the Computerized Provider Order Entry (CPOE) system. No data supported this finding in this study. It is possible that the Kardex was used in this way, but that these nurses did not view this as an important function, and thus did not report it. On the other hand, it is unlikely that features that support workarounds would be self-reported, particularly ones that suggest that nurses needed to work outside their formal scope of practice.

Although there were mixed findings, there was moderate agreement that the design of their electronic health record in their hospital did not provide a useful'status at a glance' summary, did not support prospective memory, did not aid in performing handovers, and was not sufficiently simple. There did seem to be sufficient support for some nurses concerning activity management, and, as others have found (25), handwritten paper notes (‘brains’) are still ubiquitous. The variation in the findings might result from variability in the care settings, the personal preferences of nurses for how to use EHRs, and implementation differences for EHRs.

The findings regarding the usefulness of the Kardex are not surprising in the context of similar studies of the utility of artifacts for coordination support. First, several studies have highlighted the usefulness of ‘status at a glance’ displays in complex, high-risk settings with the need to coordinate real-time interdisciplinary experts. For example, Roth et al. documented the benefits of shared large-screen displays for supervisory control in nuclear power plants (26). Similarly, ambulance dispatchers utilized a map display with Global Positioning Systems (GPS) data displaying ambulance locations (27). Likely as a result of the status at a glance map display, there was little communication regarding location between the dispatched ambulance and the dispatchers during shift change handovers. Finally, the collaborative affordances of having portable information repositories (such as on a paper sheet which contains information copied from the Kardex), shared access from multiple users at once (such as the Kardex), and a shared overview across shifts (such as the Kardex cards) have been identified as important for augmenting electronic health records (17).

Regarding the specific domain of hospital nursing, these findings suggest a path forward for incorporating what we have identified to be useful and usable about the Kardex into electronic health records. A hybrid system with information printed from an EHR can provide “clinical immediacy” in the sense of being able to immediately access information without turning a back to a patient or other staff member. Also, a paper-based system can avoid the steps and associated time with logging into a secure information repository and then navigating to where the data are displayed. This reduction in workload is particularly important, given that the introduction of electronic health records increases cognitive load due to increased information fragmentation (28). Also, when data entry is optimized by automatically pulling in updated data from the EHR, the negative finding from this study of a Kardex requiring a nurse to walk to the central unit location is avoided. Finally, standardizing the layout of the information display for multiple users was shown to be possible with the historical Kardex, even though studies of report sheets (“brains”) that are individual cognitive aids have not been. Therefore, these findings suggest that there is a willingness among nurses to standardize information displays in a structured format at the level of a hospital unit, as long as there is local control over how that structure is composed and altered over time.

There are several limitations to this study. First, the number of participants in the Kardex semi-structured interview portion was fewer than initially planned. However, we achieved theoretical saturation. Second, although we conducted the interviews recently, the Kardex is not currently used. Therefore, the insights are based upon nurses' memories of how the Kardex was used without being able to triangulate the findings through direct observations or review of actual Kardex documentation. Fourth, our validation interviews identified at least one potentially missing theme, that the Kardex supported interdisciplinary team members on the unit viewing a succinct summary of patient information at a glance. It is possible that this is because the interview questions we asked did not directly address other users of the Kardex besides the participant who was interviewed, which only included registered nurses. Finally, study participants were a convenience sample. Nevertheless, we achieved a sample with nurses with experience from multiple hospitals, in a diversity of care areas, including both adult and pediatric care, and a range of years of nursing experience and all had personal experience using the Kardex to provide clinical care in a hospital.

There are practical implications regarding our methodology. In many cases, scientific studies for digital health technologies in hospitals has been conducted solely for current technologies in use. In this paper, we have demonstrated that historical paper-based artifacts that evolved over time to be useful may provide insights for digital health innovation. Our study provides a model for how to conduct interviews that take advantage of insights generated via informal comparisons by nurses while working on features of systems no longer in use with systems that are currently in use. Many other settings have similar situations, particularly with electronic replacements of paper-based or whiteboard-based systems. We believe that this study is a useful model for informing development and design of innovative health information technology. Therefore, this comparative method could be applied to other areas where former systems are perceived to be more usable or useful than current support, as well as the opposite situation.

## Data Availability Statement

The datasets for this article are not publicly available because of ethical reasons relating to protecting the identity of nursing professionals who could potentially have a risk to reputation based upon responses to questions which inadvertently reveal their identity. Requests to discuss the content of the datasets should be directed to patterson.150@osu.edu.

## Ethics Statement

The studies involving human participants were reviewed and approved by Ohio State University Biomedical Sciences IRB. The patients/participants provided their written informed consent to participate in this study.

## Author Contributions

AM-C and KP contributed to study design, data collection, data analysis, and manuscript preparation. KE, SM-B, and EP contributed to study design, data analysis, and manuscript preparation. DW contributed to study design, literature review, and manuscript preparation. EC contributed to study design, recruitment, data collection, data analysis, and manuscript preparation. All authors contributed to the article and approved the submitted version.

## Conflict of Interest

The authors declare that the research was conducted in the absence of any commercial or financial relationships that could be construed as a potential conflict of interest.
